# Brain network topology and its cognitive impact in adult glioma survivors

**DOI:** 10.1038/s41598-024-63716-2

**Published:** 2024-06-04

**Authors:** Laurien De Roeck, Jeroen Blommaert, Patrick Dupont, Stefan Sunaert, Charlotte Sleurs, Maarten Lambrecht

**Affiliations:** 1 Department of Radiotherapy and Oncology, University Hospitals Leuven, Herestraat 49, 3000 Leuven, Belgium; 2 Department of Oncology, KU Leuven, Leuven, Belgium; 3 Leuven Brain Institute, KU Leuven, Leuven, Belgium; 4 Department of Neurosciences, KU Leuven, Leuven, Belgium; 5 Department of Imaging and Pathology, KU Leuven, Leuven, Belgium; 6 Department of Cognitive Neuropsychology, Tilburg University, Tilburg, the Netherlands

**Keywords:** CNS cancer, Cognitive neuroscience, CNS cancer, CNS cancer, Network models

## Abstract

Structural brain network topology can be altered in case of a brain tumor, due to both the tumor itself and its treatment. In this study, we explored the role of structural whole-brain and nodal network metrics and their association with cognitive functioning. Fifty WHO grade 2–3 adult glioma survivors (> 1-year post-therapy) and 50 matched healthy controls underwent a cognitive assessment, covering six cognitive domains. Raw cognitive assessment scores were transformed into w-scores, corrected for age and education. Furthermore, based on multi-shell diffusion-weighted MRI, whole-brain tractography was performed to create weighted graphs and to estimate whole-brain and nodal graph metrics. Hubs were defined based on nodal strength, betweenness centrality, clustering coefficient and shortest path length in healthy controls. Significant differences in these metrics between patients and controls were tested for the hub nodes (i.e. n = 12) and non-hub nodes (i.e. n = 30) in two mixed-design ANOVAs. Group differences in whole-brain graph measures were explored using Mann–Whitney U tests. Graph metrics that significantly differed were ultimately correlated with the cognitive domain-specific w-scores. Bonferroni correction was applied to correct for multiple testing. In survivors, the bilateral putamen were significantly less frequently observed as a hub (*p*_*bonf*_ < 0.001). These nodes’ assortativity values were positively correlated with attention (*r*(90) > 0.573, *p*_*bonf*_ < 0.001), and proxy IQ (*r*(90) > 0.794, *p*_*bonf*_ < 0.001). Attention and proxy IQ were significantly more often correlated with assortativity of hubs compared to non-hubs (*p*_*bonf*_ < 0.001). Finally, the whole-brain graph measures of clustering coefficient (*r* = 0.685), global (*r* = 0.570) and local efficiency (*r* = 0.500) only correlated with proxy IQ (*p*_*bonf*_ < 0.001). This study demonstrated potential reorganization of hubs in glioma survivors. Assortativity of these hubs was specifically associated with cognitive functioning, which could be important to consider in future modeling of cognitive outcomes and risk classification in glioma survivors.

## Introduction

Gliomas are the most common type of primary brain tumors^1^, arising from glial cells and inherently invading brain tissue. The treatment for these tumors entails surgery and/or chemoradiotherapy, where combined treatment is mostly indicated. One of the most elusive long-term sequelae related to both the tumor and its treatment following therapy in glioma patients is cognitive dysfunction^2–4^. Deterioration in cognitive functioning, including memory loss and concentration disorders, places a heavy burden on both the medical and socio-economic aspects of the patient’s lives. Cognitive dysfunction is a complex behavioral symptom, which cannot be solely attributed to damage to a focal brain region^5,6^. Therefore, in recent years, the lingering cognitive impact has increasingly been attributed to an orchestration of multiple neural structures, warranting to approach cognitive processes from a network point of view^7^.

In this context, graph theory has emerged as a recent framework in neuroscience for modelling brain connectivity patterns that could underlie cognitive processes^8^. A brain network or so-called connectome can be constructed using different modalities. Electroencephalography (EEG), magnetoencephalography (MEG) and functional MRI (fMRI) can be used to model functional brain networks and diffusion-weighted MRI for structural networks^9,10^. In a network, nodes represent distinct brain regions, and edges between the nodes correspond to a selected measure of connectivity. In case of a structural brain network, connectivity values are estimated based on the underlying white matter tracts connecting these regions^10^. One crucial aspect of neural networks is the presence of so-called "hubs", which play a central role in information processing and integration^8^. Damage to these hubs could consequently result in a disproportionate failure of the network’s connectivity, resilience, and robustness^11^. Moreover, as these hub regions have high metabolic demands, they are specifically vulnerable to diseases such as brain tumors^12–14^ and neuro-degenerative and -psychiatric conditions^15–17^, highlighting them as potential targets of neurotoxicity and brain pathology.

Given the complex interplay between glial tumor cells and the surrounding brain cells, gliomas render the ultimate network disease^18^. Previous studies have reported alterations in both functional and structural brain networks in this population, although inconsistencies exist across studies regarding the direction and magnitude of effects^19^. Most studies addressed functional network alterations by using MEG data^20–23^ or functional MRI^24–28^, reporting altered integration and segregation of the network of glioma patients, both in the lesioned and contralateral hemisphere. Structural network changes have been observed in measures of integration, segregation and centrality, both peripheral as well as in areas surrounding the tumor^13,29–34^.

Only few studies investigated the role of structural hubs in cognitive processes to date, with a main focus on childhood gliomas^13^ and infratentorial tumors^35^. These studies showed alterations in hub-specific integration and segregation as well as local efficiency, which played a role in executive functioning and intelligence outcomes, respectively.

In sum, the role of structural hubs in cognitive functioning is poorly investigated. Therefore, the field requires a more defined conceptual approach that considers glial tumors and the accompanying brain damage as a network disease. This entails developing a conceptual framework that elucidates how localized network disruptions may give rise to global or hub-related network alterations and the link with cognitive processes. By examining brain network properties more in-depth, cognitive impairment that a patient may face could be better predicted in the future.

In this study, we explored structural network alterations in glioma patients after treatment and more specifically the role of hubs in cognitive functioning. First, we addressed whether hub locations and cognitive outcomes differed between glioma patients and healthy controls. Secondly, we studied the association of whole-brain and hub-specific nodal graph metrics with cognitive functioning.

## Materials and methods

### Participants and study design

This cross-sectional study consecutively enrolled Dutch-speaking adult (≥ 18 year) patients treated for a WHO grade 2 or grade 3 glioma between 2007 and 2019 (at least one year post-surgery and/or chemoradiotherapy) at the University Hospitals of Leuven (Belgium), conform to CONSORT 2010 guidelines. Patients were screened based on a medical database. All patients in routine clinical follow-up with a WHO performance status of 0 to 2 were eligible, and recruited and scanned between 2021 and 2022.

Exclusion criteria included any prior diagnosis of psychiatric conditions, intellectual disability, a genetic syndrome (e.g., Down), relapse after chemo- and/or radiation therapy, any other brain neoplasms (e.g., meningioma, metastases) or MR contraindications.

Healthy controls were recruited via online forums and were matched at individual level for age (maximal age difference of 3 years), sex and education.

All participants completed a self-report inventory, a cognitive test battery and neuroimaging protocol, of which the latter two were completed on the same day. This study was approved by the Ethical Committee of the University Hospitals of Leuven (S63580) and performed in accordance with the declaration of Helsinki. All included participants provided their written informed consent.

### Cognitive data

Cognitive performance was assessed with a one hour assessment battery of behavioral tests including: digit span, digit symbol substitution and matrix reasoning subtest of the Wechsler Adult Intelligence Scale, fourth edition (WAIS IV)^36^; the Trail Making Test (TMT) part A & B^37^; the Hopkins Verbal Learning Test-Revised (HVLT-R)^38^; the Controlled Word Association Test (COWAT)^39^; the Stroop Color and Word Test (SCWT)^40^; and the Grooved Pegboard test^41^.

Raw cognitive assessment scores were transformed into w-scores, which are analogous to z-scores but adjusted for specific covariate(s) to account for the large variability in the sample, i.e. age (range: 21–70 years) and education (primary school-university) in this study. Based on a linear regression model, age- and education specific (ISCED 2011 definition) regression coefficients were calculated based on the healthy controls dataset and obtained test scores were then subtracted from the estimated scores^42^.

As longer response times indicate worse performance, w-scores for time-critical tests were inverted (multiplied by − 1) for consistency in interpretation (i.e. higher w-score indicates better performance). Subsequently, these test scores were categorized into six main cognitive domains (Table 1) based on the DSM-V definition of neurocognitive functioning and between-test correlations^43^. In specific, the matrix reasoning subtest of the WAIS IV was used as a proxy measure of premorbid intelligence. The domain summary scores were calculated for all participants by averaging the test-specific w-scores of that particular domain^44^. Cognitive impairment was defined based on the International Cancer and Cognition Task Force recommendations, as two or more test scores with w-scores at or below − 1.5 or at least one test score with a w-score at or below − 2.0^45^.

**Table 1:** Cognitive (sub)test grouped per cognitive domain.

Cognitive domain	Neurocognitive test	Outcome measurement
Memory	HVLT-R immediate recall	Sum score—learning
	HVLT-R delayed recall	Sum score
	HVLT-R recognition	Good recognition-mistakes
Executive functioning	TMT B	Time
	SCWT interference	Interference score
	WAIS IV digit span backwards	Total number of series
	WAIS IV sequencing	Total number of series
Attention / processing speed	WAIS IV symbol substitution	Sum score
	TMT A	Time
	SCWT colors	Time
	SCWT words	Time
	WAIS IV digit span forward	Total number of series
Motor function	Grooved pegboard	Time (non)dominant hand
Proxy IQ	WAIS IV matrix reasoning	Items correct
Language	COWAT semantic	Sum of words
	COWAT phonemic	Sum of words

HVLT-R, Hopkins Verbal Learning Test Revised; TMT, Trail Making Test; SCWT, Stroop Color Word Test; WAIS IV, Wechsler Adult Intelligence Scale, fourth edition; IQ, intelligence quotient; COWAT, Controlled Oral Word Association Test.

### Image acquisition

Magnetic resonance (MR) images were acquired on a Philips Achieva scanner operating at 3 T with a 32-channel phased-array head coil. First, T1-weighted images (MPRAGE) were acquired (7 min, voxel size = 0.800 mm^3^ isotropically, FA = 8°, TR/TE = 5.800/2.500 ms, FOV = 320 × 320 voxels, 208 slices), followed by a 4-min 3D FLAIR scan (voxel = 1 mm^3^ isotropically, TI = 165 ms, FA = 90°, TR/TE = 4800/340 ms, NSA = 2, FOV = 256 × 256 voxels, 183 slices). Multi-shell diffusion-weighted images were acquired with two phase encoding directions = AP (8 min) and then PA (8 min), voxel size = 2 × 2 × 2mm^3^, FA = 90°, TR/TE = 5000/80 ms, FOV = 112 × 112 × 72 voxels, b-values = 0/700/1200/2800 s/mm^2^ with a total of 10/24/40/76 gradient directions respectively, SENSE = 2.500, and multiband = 2. Other acquired sequences (T2-weighted images, Arterial Spin Labeling, resting-state functional MRI, Quantitative Susceptible Mapping and Susceptibility Weighted Images) are beyond the scope of this manuscript.

### Image preprocessing

**Figure 1:**
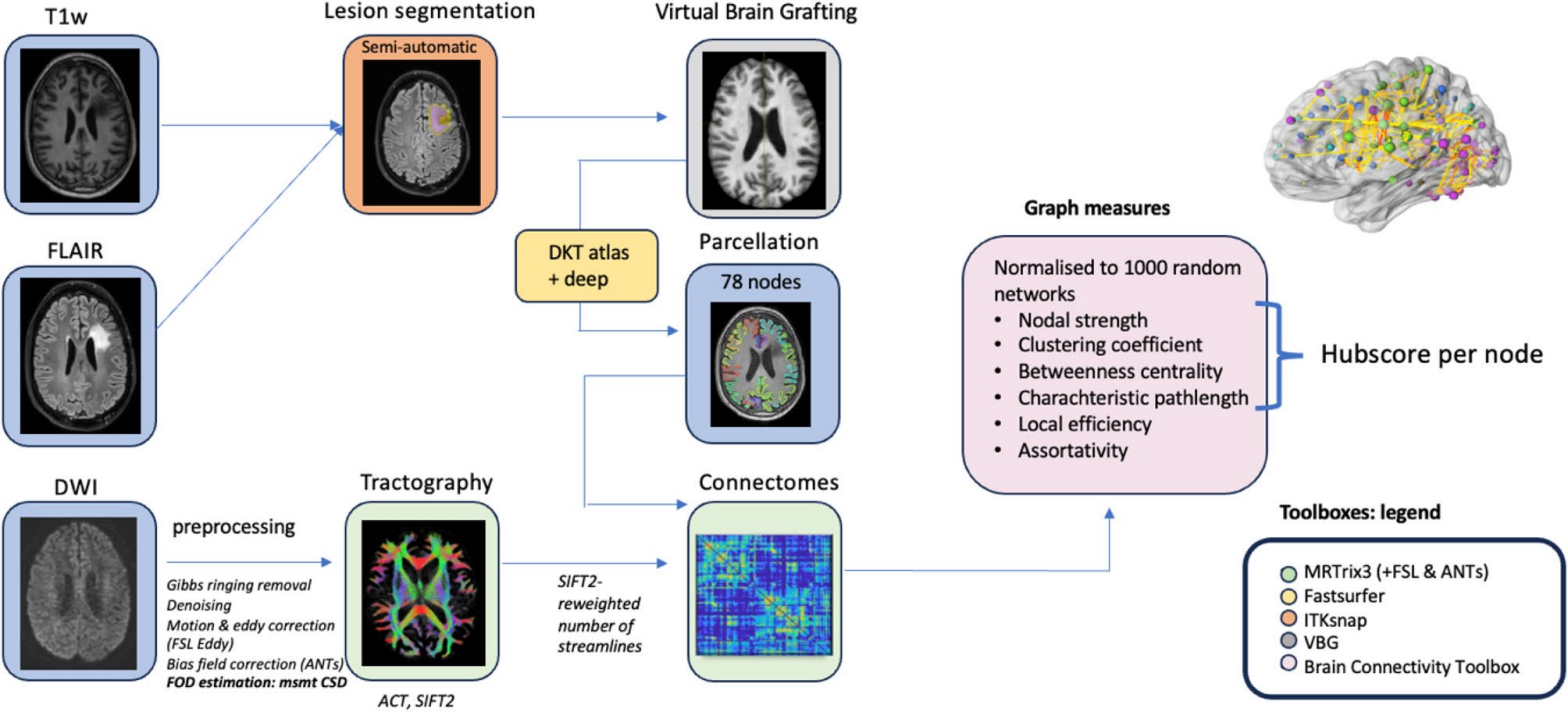
Imaging processing pipeline. T1w: T1-weighted images, FLAIR: Fluid-attenuated inversion recovery images, DWI: diffusion-weighted images, FOD: fibre orientation distribution, msmt CSD: multi-shell multi-tissue Constrained Spherical Deconvolution, ACT: anatomically-constrained tractography, DKT: Desikan-Killiany-Tourville atlas.

Image processing was performed using Matlab-based scripts (Matlab R2023a and Bash) and validated toolboxes. An overview of the imaging preprocessing and analysis is depicted in Fig. 1.

First, semi-automatic lesion segmentation of resection cavity, tumoral tissue and/or perilesional gliosis, and ventricles was performed by using resseg^46^, HD-glio-auto^47^ and FastSurfer^48^, respectively. Subsequently, all lesions were manually corrected in ITK-snap (v3.6.0)^49^ by a radiation-oncologist (LDR). Lesion-free and T1-weighted images and masks were created using Virtual Brain Grafting (VBG v0.61^50^). The VBG-corrected anatomical scan was then used to parcellate subcortical regions (thalamus, caudate, putamen, pallidum, amygdala, hippocampus, and accumbens area) and the cortex according to the Desikan-Killiany-Tourville (DKT) atlas using FastSurfer (v2.0^48^), which resulted in 78 cortical parcellations^51,52^. An affine transformation followed by a nonlinear registration (mutual information cost-function) was done using ANTs (v2.3.5^53^) to transform these parcellations to the mean fiber orientation distribution (FOD) image.

DWI preprocessing included Gibbs-ringing artifact removal^54^ and denoising^55^ using MRtrix3 (v3.0^56^,), eddy current, EPI distortion and motion correction^57^ using FSL (v6.0.1) Eddy, and N4 bias-field correction^58^ using ANTs^53^. Links to the used software can be found in Supplementary Materials.

For each subject, tissue-specific constrained spherical deconvolution (CSD) response functions and whole-brain tractograms (iFOD2, ten million fibers, minimum/maximum length = 5/300 mm, max. angle = 45°, FOD amplitude threshold 0.06, dynamic seeding with seed-based termination), were constructed using multi-shell multi-tissue CSD (with a maximum of spherical harmonic degree Lmax = 8), and anatomically-constrained tractography with SIFT2 re-weighting of the streamlines (10 million fibers) in MRTrix3 (v3.0^56^)^59,60^. The T1-weighted images (including parcellations and lesions) were aligned to FOD space, which was visually checked.

### Connectome construction and graph metrics

By combining the parcellations generated by FastSurfer (78 nodes) and the whole-brain, SIFT2 re-weighted tractograms, structural connectomes were generated for each subject. Self-connections and floating nodes (a singular node in nine out of 50 patients, varying among subjects) were removed from the subject-specific connectomes^61^. Floating nodes were defined as nodes in which less then 1000 out of 10 million streamlines arrived. Edge (streamline count) weights were normalized by dividing them by the sum of all edge weights.

Weighted graph measures (based on this weighted graph) of nodal strength, shortest path length, betweenness centrality, local efficiency, assortativity and clustering coefficient were calculated for all 78 nodes, together with, whole-brain graph measures of clustering coefficient, local efficiency, characteristic path length and global efficiency. All measures were calculated using the Brain Connectivity toolbox (v2019-03-03^62^) and an in-house developed MATLAB script. We opted to include these specific graph measures as nodal strength, clustering coefficient, betweenness centrality, and shortest path length are essential for calculating the hubscore^63^. Moreover, we’ve added local efficiency and assortativity as they have been previously associated with cognition in neuro-oncological research^24,35^.

In brief, characteristic path length quantifies the average shortest path length across all nodes. Shortest path length between two nodes is the path with minimal cost. The shortest path of a node is the average of the shortest paths from that node to any other node in the network. It provides insights into the overall efficiency of information or signal propagation in a network and was calculated by the algorithm of Dijkstra^64^.

Clustering coefficient measures the degree of local clustering or connectivity within a network. It quantifies how well-connected the neighbors of a particular node are to each other. The global clustering coefficient is the average of the clustering coefficients across nodes. For the calculation of the clustering coefficient, we used the generalization for weighted networks proposed by Wang et al.^65^.

Local efficiency measures how efficiently information can be exchanged or transferred among the immediate neighbors of a node. A high local efficiency indicates that the node’s neighbors are well-connected to each other and can quickly share information. The local efficiencies were calculated as recommended by Wang et al.^65^.

Global efficiency is a measure of how well-connected and interconnected a network is as a whole. Note that global efficiency should not be confused with the average local efficiency since it is the average of the efficiencies across nodes. Efficiency of a node is defined as the average of inverse “distances” between the node and any other node in the network.

Nodal strength is a measure of the cumulative influence or importance of a node within a network, taking into account the strength of its connections to other nodes. Nodes with higher nodal strengths are considered more central or influential within the network because they have stronger interactions with other nodes.

Betweenness centrality measures how often a node occurs on the shortest paths between any two other nodes, making it a key metric for identifying nodes that play a crucial role in facilitating information flow.

Assortativity measures the tendency of nodes in a network to connect to other nodes with similar or dissimilar properties.

Since graph measures depend on the weight distribution, we normalized the whole-brain and nodal graph measures: 1000 equivalent random graphs (i.e., with the same number of nodes and the same weight distribution) were generated by randomly permuting the edges, while taking into account the self-connections (no weight) and the connectome symmetry and removing graphs with floating nodes. Next, the graph measures were divided by the corresponding median values obtained from the random networks in order to scale the observed measures relative to what is expected in random networks with the same weight distribution. We opted to use the median value of the random networks instead of the mean for normalization, since the median is not influenced by outliers. The code is available on the Open Science Framework (https://osf.io/7t3xu/).

### Hub identification

For each node an overall hubscore was calculated based on its nodal strength, betweenness centrality, clustering coefficient, and shortest path length^63^. Nodes reaching the highest 20% of betweenness centrality and nodal strength values and lowest 20% of shortest path length and clustering coefficient values, were assigned one point for each criterion if true, resulting in the overall hubscore (0–4). Nodes with a total score of 2 or more in > 80% of subjects per group were defined as network hubs^63^. A non-hub was defined as a node with a hubscore of zero in every healthy control.

### Statistical analyses

First, group differences in domain-specific cognitive w-scores and whole-brain graph measures (local efficiency, global efficiency, characteristic path length and clustering coefficient) were explored using non-parametric Mann–Whitney U tests. To address whether the hub regions differed in frequency between both groups, a Chi-Square Test was conducted.

Second, differences in nodal graph metrics between patients and controls were tested for the hub nodes and non-hub nodes separately in two mixed-design ANOVAs given that these parcellated regions are within-subjects measurements. In case of non-normality of the graph measures, the measures were log-transformed before conducting the analysis. When the assumption of sphericity was violated, a Greenhouse Geisser correction was applied.

Next, to explore the associations between log-transformed graph measures and cognitive outcomes, graph measures that were significantly different between groups in both hubs and non-hubs were correlated with the w-scores per cognitive domain (*n* = 6) using Pearson correlation coefficient. The McNemar’s test was used to assess whether correlations occurred more frequently in hubs compared to non-hubs. Moreover, to explore potential differences in brain networks due to the local disturbing effect of tumoral involvement of the putamen, a subgroup analysis was performed in patients without involvement of the putamen. The significance threshold for statistical tests was set at *α* < 0.050. Bonferroni correction for multiple comparisons was applied when exploring (a) group differences in six cognitive domain w-scores (α < 0.050/6) and (b) in four whole-brain graph measures (*α* < 0.050/4), (c) when correlating six cognitive w-scores to the significantly different whole-brain (*α* < 0.050/24) and nodal graph measures (see below), and (d) when comparing the relative frequencies of associations that are significant between cognitive scores and (four) nodal graph measures in hubs versus non-hubs (*α* < 0.050/4). All statistical analyses were conducted using SPSS (version 28.0.1.1). All analyses were repeated on weighted non-normalized graphs and binary graphs of different densities to assess robustness of our results (Supplementary Materials).

## Results

### Participants

**Figure 2:**
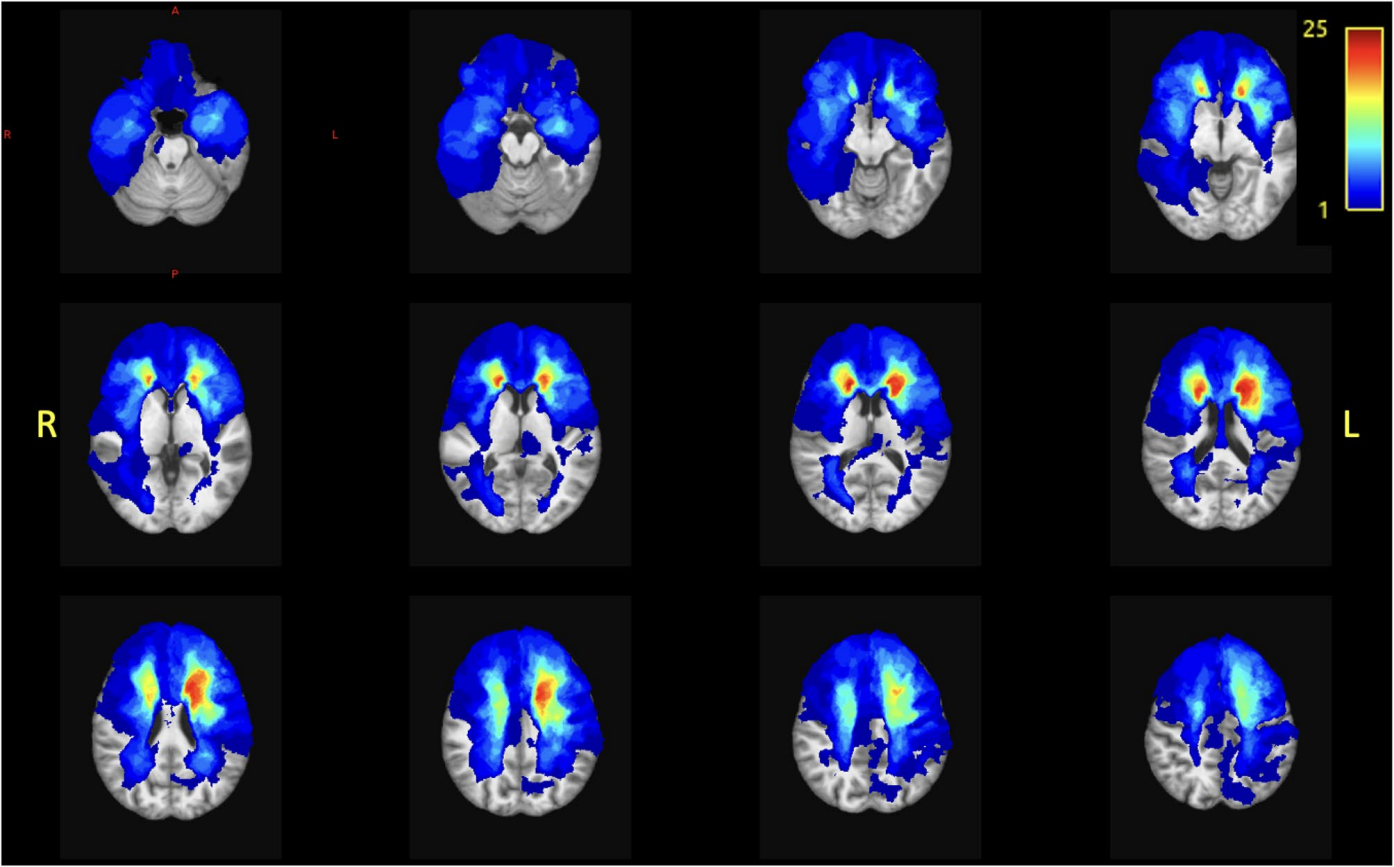
Heatmap of glioma lesions. Heatmap of glioma lesions including resection cavity, rest tumor and surrounding gliosis (voxel-based), showing most lesions (n = 25) in the left frontal lobe.

A total of 397 patients underwent screening. Among the 60 eligible patients, 50 patients participated in the study. Clinical data & demographics of all participants are summarized in Table 2. Age (mean = 42 years), sex (50% male) and education (ISCED 2011 high education = 55%) were equally distributed in both groups. For glioma patients, time between last treatment and assessment was 5 years on average. Most patients were diagnosed with a WHO grade 2 (54%) oligodendroglioma (60%) and received multimodal treatment: surgery (86%), radiotherapy (76%) and chemotherapy (70%). A heatmap of lesion overlap is presented in Fig. 2. Lesions were mostly located in the left frontal lobe (in 25 patients) and more specifically, in the bilateral inferior and medial frontal gyrus, and in the left superior frontal gyrus.

**Table 2:** Descriptive characteristics of the participants.

Characteristics	Patients (n = 50)	Controls (n = 50)
Demographics		
Age (in years)		
*Mean* (SD)	42.58 (13)	42.42 (13)
*Median* (range)	39 (23–70)	40 (21–72)
Sex: females, *n* (%)	25 (50)	25 (50)
Education (Verhage categories)^66^		
Level 3, *n* (%)	1 (2)	1 (2)
Level 4, *n* (%)	8 (16)	8 (16)
Level 5, *n* (%)	8 (16)	8 (16)
Level 6, *n* (%)	16 (32)	16 (32)
Level 7, *n* (%)	17 (34)	17 (34)
Handedness		
Right,* n* (%)	42 (84)	47 (94)
Left, *n* (%)	5 (10)	3 (6)
Both, *n* (%)	3 (6)	0 (0)
Anti-epileptic drugs usage		
Yes,* n* (%)	33 (66)	0 (0)
Tumor treatment		
Time since last treatment in years, *mean* (SD)	5 (3,5)	
Surgery, *n* (%)	43 (86)	
Radiotherapy, *n* (%)	38 (76)	
Total dose		
54 Gy, *n* (%)	20 (53)	
59.4 Gy, n (%)	7(18)	
60 Gy, *n* (%)	11 (29)	
Chemotherapy, *n* (%)	35 (70)	
PCV, *n* (%)	23 (46)	
TMZ, *n* (%)	12 (24)	
Tumor location*		
Frontal, *n* (%)	35 (70)	
Parietal, *n* (%)	6 (12)	
Temporal, *n* (%)	15 (30)	
Occipital, *n* (%)	1 (2)	
Brainstem, *n* (%)	2 (4)	
Thalamus, *n* (%)	1 (2)	
Involved hemisphere		
Left, *n* (%)	31 (62)	
Right, *n* (%)	18 (36)	
Both, *n* (%)	1 (2)	
Lesion volume (cm^3^)		
Median (range)	52.24 (1.87–162.19)	
Tumor characteristics		
Histology (WHO 2016)		
Oligodendroglioma, *n* (%)	30 (60)	
Diffuse astrocytoma, *n* (%)	16 (22)	
Anaplastic astrocytoma, *n* (%)	2 (4)	
Anaplastic oligodendroglioma, *n* (%)	2 (4)	
WHO grade		
2, *n* (%)	27 (54)	
3,* n* (%)	23 (46)	
IDH mutation		
IDH1-mutation, *n* (%)	24 (48)	
IDH1- wild type, *n* (%)	5 (10)	
IDH1, NOS, *n* (%)	21 (42)	

*Overlap of multiple brain tumor locations possible. Gy, Gray; PCV, procarbazine, lomustine (CCNU) and vincristine; TMZ, temozolomide; WHO, World Health Organization; IDH, Isocitrate dehydrogenase; NOS, not otherwise specified.

### Cognitive data

**Figure 3:**
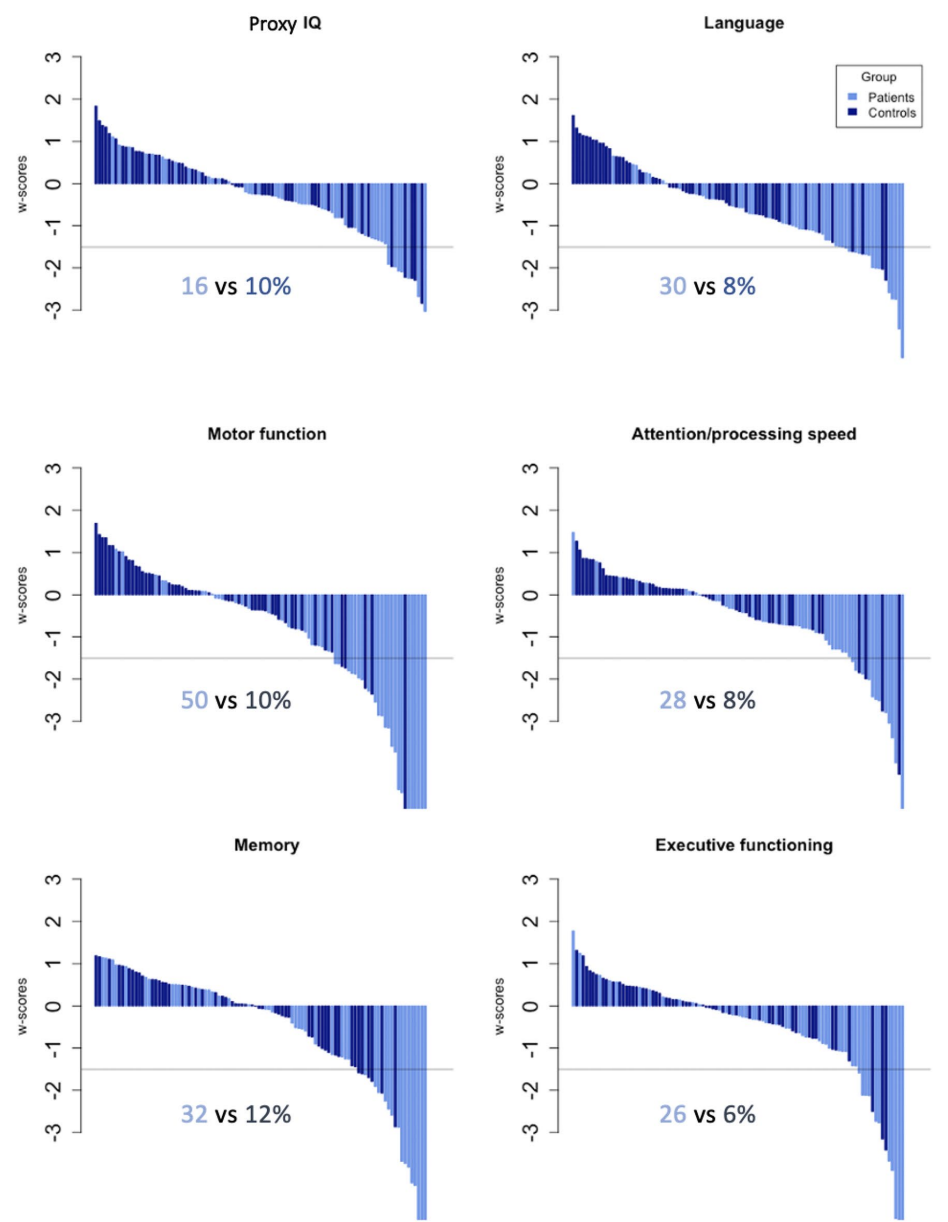
Waterfall plots per cognitive domain. Waterfall plots: X-axis: each bar represents an individual (light blue: patients, dark blue: controls). Y-axis: w-scores with cut-off of a w-score ≤ − 1.50 for impairment. Per cognitive domain the percentage of impaired subjects (light blue for patients and dark blue for controls) is indicated below the plot.

Overall, cognitive impairment was observed in 60% of patients compared to 10% of the healthy controls (Fig. 3). Patients performed significantly worse on each cognitive domain, including memory (U = 1710, *p*_*bonf*_ = 0.002); executive functioning (U = 1828, *p*_*bonf*_ < 0.001); attention/processing speed (U = 2027, *p*_*bonf*_ < 0.001); motor functioning (U = 1999, *p*_*bonf*_ < 0.001); language (U = 2059, *p*_*bonf*_ < 0.001) and proxy IQ (U = 1719, *p*_*bonf*_ < 0.001).

### Graph theory analyses

#### Whole-brain graph measures

All four whole-brain graph measures differed significantly between both groups. Significantly higher clustering coefficient (*p*_*bonf*_ < 0.001, U = 163) and local efficiency (*p*_*bonf*_ < 0.001, U = 353), a higher characteristic path length (*p*_*bonf*_ < 0.001, U = 415) and lower global efficiency (*p*_*bonf*_ < 0.001, U = 2188) were found in patients compared to healthy controls.

#### Hub-specific findings

**Figure 4:**
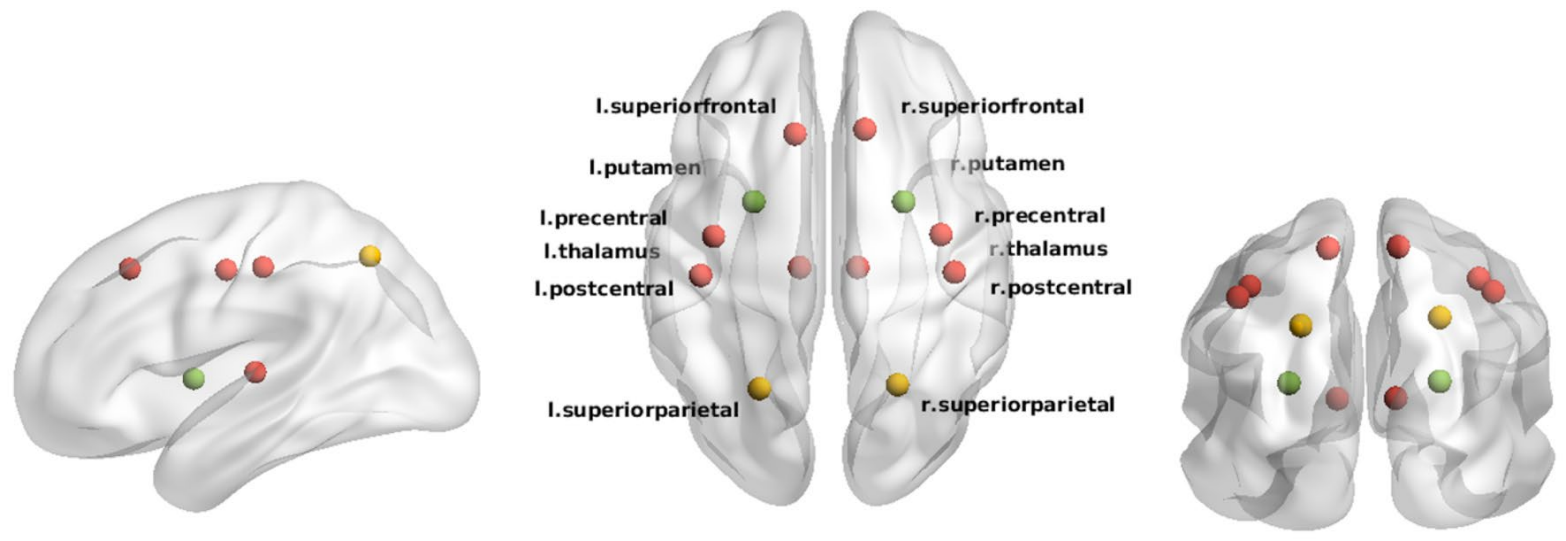
Hub regions (12 nodes) defined in healthy controls. Nodes defined as hub in both patients and controls are indicated in red. Nodes indicated in yellow (left/right superior parietal) and green (left/right putamen) have a lower likelihood of being a hub in patients than in controls (green: significant difference in frequency between groups, yellow: no significant difference in frequency between groups).

In controls, 12 out of 78 nodes were classified as network hubs (Supplementary Table S1 and Fig. 4). In patients, the left and right superior parietal gyri and the left and right putamen were less frequently defined as hub, however only reaching statistical significance for the left and right putamen (*p*_*bonf*_ < 0.001 and *p*_*bonf*_ = 0.046, respectively).

Thirty out of 78 nodes were defined as pure non-hubs in controls. In patients, two left-sided nodes (pars orbitalis and paracentral gyrus) and five right-sided nodes (entorhinal, isthmus cingulate, paracentral, pericalcarine and posterior cingulate gyrus) could not be identified as pure non-hubs, which might indicate network reorganization (Supplementary Table S2).

The floating nodes which have been removed from the connectomes in 9 out 50 patients were never identified as a hub nor a non-hub, resulting in an equal number of included nodes for each subject.

#### Mixed-design ANOVA for nodal graph measures

All statistical findings of the ANOVA comparing the nodal graph metrics between groups, are summarized in Table 3. This analysis showed that participant groups exhibited significant differences in betweenness centrality in non-hubs (F(1,98) = 3.620, *p*_*bonf*_ < 0.001, ηp^2^ = 0.272), with lower centrality in patients compared to controls (p_bonf_ < 0.001). No centrality differences were encountered in hubs (F(1,98) = 0.929, *p*_*bonf*_ = 0.338, ηp^2^ = 0.099).

**Table 3:** Between subjects ANOVA results per nodal measure for hubs and non-hubs.

Nodal graph measure	Node group	df	df (error)	F-value	*p*_bonf_	ηp^2^
Local efficiency	Hubs	1	98	20.138	< 0.001	0.170
	Non-hubs	1	98	26.195	< 0.001	0.211
Assortativity	Hubs	1	98	53.510	< 0.001	0.353
	Non-hubs	1	98	51.259	< 0.001	0.360
Clustering coefficient	Hubs	1	98	133.680	< 0.001	0.577
	Non-hubs	1	98	87.937	< 0.001	0.473
Nodal strength	Hubs	1	98	1.702	0.195	0.017
	Non-hubs	1	98	2.800	0.970	0.028
Betweenness centrality	Hubs	1	98	0.929	0.338	0.099
	Non-hubs	1	98	3.620	< 0.001	0.272
Shortest path length	Hubs	1	98	69.455	< 0.001	0.415
	Non-hubs	1	98	18.702	0.001	0.163

Both in hubs and non-hubs shortest path length, local efficiency, assortativity and clustering coefficient differed significantly between groups. Regarding shortest path length, a significant main effect of group was found both in non-hubs (F(1,96) = 18.702, *p*_*bonf*_ = 0.001, ηp^2^ = 0.163) and hubs (F(1,98) = 69.455, *p*_*bonf*_ < 0.001, ηp^2^ = 0.415).

Higher nodal shortest path length, local efficiency, and clustering coefficients were found in patients compared to controls (*p*_*bonf*_ ≤ 0.001). Specifically in hubs, lower assortativity was observed in patients compared to controls (*p*_*bonf*_ ≤ 0.001).

No statistically significant main effect of group between subjects was found for nodal strength, neither in hubs nor in non-hubs. Except for nodal strength and betweenness centrality in hubs, large effect sizes were observed (ηp^2^ > 0.140). A summary of the nodal measures is displayed in Supplementary Table S3, together with the results of the within-subjects effects (across within-subject nodes) in Supplementary Table S4. Since intracranial volume (ICV) could affect the CSD measures, we repeated the analysis while correcting for ICV showing robust findings (Supplementary Table S5).

Post-hoc validation analyses in only a subgroup of patients without tumor in the putamen (n = 14) showed robust findings, with significant group differences in all four whole-brain measures (p < 0.001). Similar to the findings in the whole sample (n = 50), nodal measures of shortest path length, local efficiency, assortativity and clustering coefficient differed significantly between groups in both hubs and non-hubs, while group differences were only encountered in non-hubs for betweenness centrality. In contrast with our findings in the whole sample, patients without tumoral invasion of the putamen did show significant group differences in nodal strength in both hubs (*p* = 0.021) and non-hubs (*p* < 0.001).

### Associations between cognitive domain scores and graph measures

#### Whole-brain graph measures

Whole-brain graph measures of clustering coefficient, global and local efficiency correlated positively with proxy IQ w-scores (*r*(98) = 0.690, 0.570 and 0.500, respectively, *p*_*bon*f_ < 0.001). No significant correlations could be found for other cognitive domains, nor for the whole-brain graph measure of characteristic path length.

#### Nodal graph measures

Nodal graph measures that differed significantly between groups in both hubs and non-hubs (i.e. clustering coefficient, local efficiency, shortest path length and assortativity) were correlated with the cognitive w-scores per cognitive domain (*n* = 6) and per node (12 hubs & 30 non-hubs). Significant correlations (*α* < 0.050/1008) are summarized in Supplementary Table S6.

#### Hubs vs non-hubs

**Figure 5:**
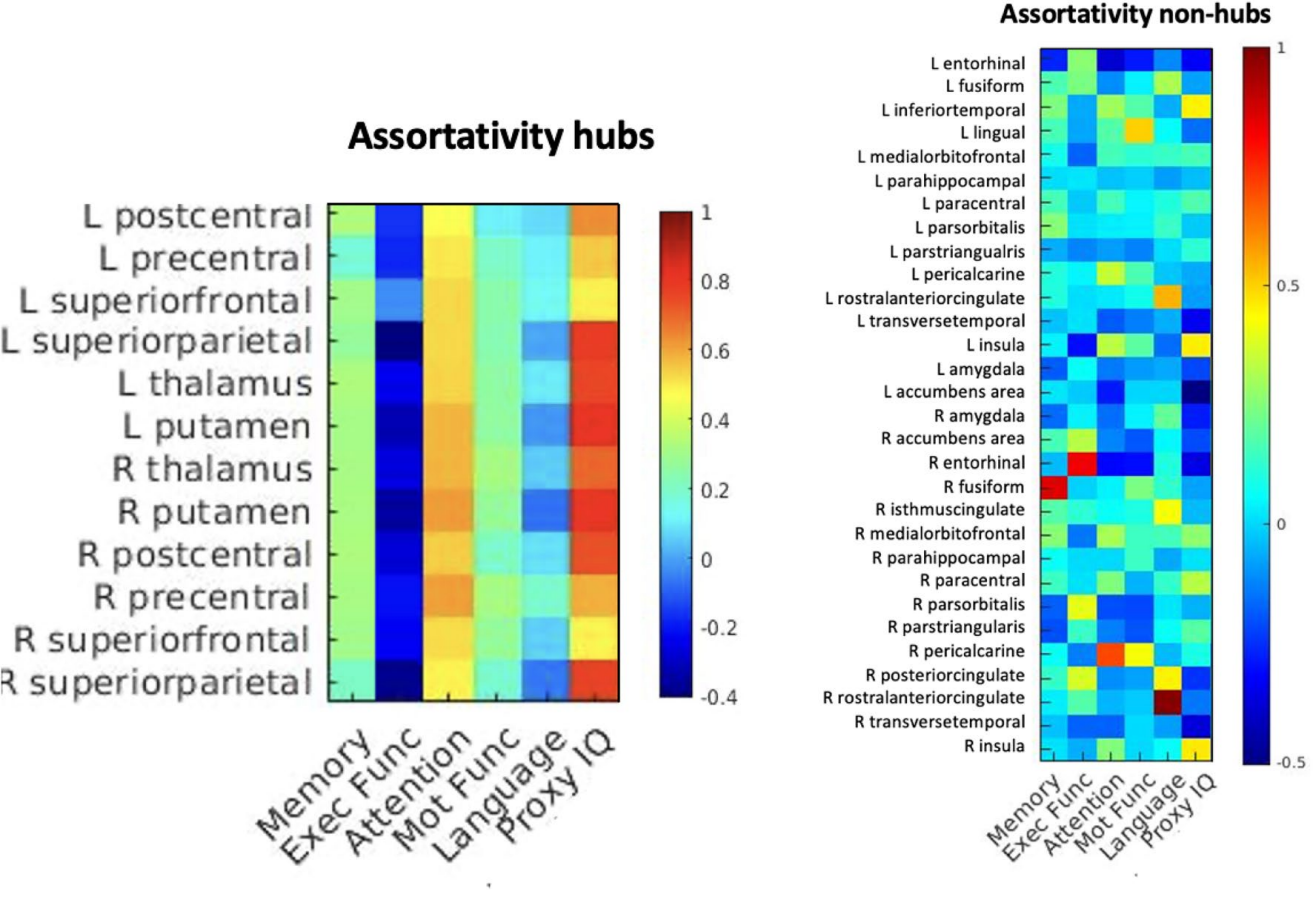
Correlation matrices of cognitive outcomes (n = 6) per node in hubs and non-hubs for nodal measure of assortativity.

Attention and IQ domain scores were consistently and highly correlated with all (100%) assortativity values of the hub areas (*r*(98) > 0.463, *p*_*bonf*_ < 0.001). By contrast, this encountered association was mostly inconsistent in non-hub areas (37%), which correlated with diverse cognitive domains (Fig. 5).

Overall, clustering coefficient, shortest path length and assortativity yielded significant correlations with cognitive outcomes, occurring more often in hubs compared to non-hubs. However, the impact of hubness was only statistically significant for assortativity (*p*_*bonf*_ < 0.001), meaning the node’s assortativity correlated significantly more often with cognitive outcomes in hubs compared to non-hubs (Supplementary Table S7 and Figure S1,S2 and S3).

All analyses were repeated once more for the binary networks of different densities. Results are depicted in the Supplementary Materials.

## Discussion

In this study, we investigated the role of structural brain networks and hub topology on cognitive outcomes in glioma survivors. Across the entire brain, we found the network of glioma patients to be less integrated and showed higher segregation, which was associated with proxy IQ. On nodal level, twelve nodes were identified as hubs in healthy controls, with the bilateral putamen being significantly less frequently designated as hubs in glioma patients. Furthermore, some nodal metrics of clustering coefficient, assortativity and shortest path length exhibited correlations with cognitive outcomes. However, when comparing hubs and non-hubs, particularly the assortativity of hubs seemed of importance in cognitive outcomes.

Previous clinical studies have already illustrated structural reorganization of the brain’s network in glioma patients, both in the contra- and ipsilesional hemisphere^67,68^. These topological network alterations are not confined to local regions but extend throughout the entire brain. In high-grade gliomas, this global re-organization could be fostered by glutaminergic tumor-neuron synapses, which allow glial tumor cells not only to promote therapeutic resistance and tumor growth, but also to communicate extensively with and to invade the surrounding brain network^69^. However, evidence on the contribution of these tumor-neuron synapses on the network organization in oligodendrogliomas is lacking and the exact mechanisms at the cellular level yet to be studied in future studies. Nonetheless we observed an altered whole brain topology showing higher segregation (local efficiency and clustering coefficient) and lower global efficiency of the network in glioma patients, compared to controls. In other words, the brain’s network in this cohort of glioma patients showed lower overall efficiency in information transfer, but also local restructuring into more specialized subnetworks. We found this reduced global efficiency to correlate with lower performance on intellectual tasks, which is in accordance with findings in healthy individuals^70^ and in functional network studies^71^.

Besides altered whole brain connectivity, we also demonstrated changes of the local topology in this population. Patients’ regional networks showed longer path length, lower betweenness centrality, lower assortativity, and greater segregation (local efficiency and clustering coefficient) compared to healthy controls. This higher segregation represents the most consistent observation in network studies involving glioma patients, indicating a tendency of brain tumors to interfere with long-range connections between nodes. And more specifically, the hubs showed the tendency to become less connected to nodes with the same characteristics (i.e. higher nodal strength), reflecting in lower assortativity. This higher assortativity—as measured in hubs- has been linked to increased vulnerability to random attacks^72–74^. Given that patients demonstrate lower assortativity in hubs compared to controls, it implies that neurotoxic treatments could potentially disrupt the coherence of hubs, thereby leading to cognitive symptoms.

Ahead of their time, Bullmore and Sporns^8^ defined such nodes central in the network as these so-called hubs. In this study, we identified twelve hub regions in healthy controls that were symmetrically distributed and consistent across both groups. Our hypothesis posited that the topology of these hub regions would be altered in glioma patients compared to controls and that these changes would hold significance in relation to cognitive functioning. Notably, we found the left and right putamen to be significantly less frequently defined as a hub in patients compared to controls. This re-organization of hub regions has been observed in previous structural network studies ass well, both in the contralateral and ipsilesional hemisphere^29,67,71^. However, the clinical significance of these findings remains rather unexplored. Douw et al.^67^ found higher hub-hub connections in the contralateral hemisphere of glioma patients, which correlated with overall performance status. In various neurological diseases, these hubs have been recognized as key player in cognitive processes^75,76^. However, in the case of glioma patients, this potential link has received far less attention^13,24,35^.

In this study, we found nodal measures of assortativity, clustering coefficient, and shortest path length to be more frequently associated with cognitive outcomes in hubs compared to non-hub regions, however only reaching statistical significance for assortativity. Therefore, it appears that this clustering of specific highly central nodes (or hubs) may hold a pivotal role in hub failure and cognitive outcomes. These findings align with earlier findings of Sleurs et al.^35^, who observed that local efficiency was associated with intelligence scores, particularly within hubs in infratentorial tumor survivors. Additionally, we found a strong and statistically significant correlation between higher assortativity and better performance on attention and intelligence tasks across all twelve hubs in our study, which is consistent with the findings of functional network studies^24^. Structural nodal hub-related graph metrics and assortativity in particular might thus be important to consider in predicting cognitive outcomes in glioma survivors. To the best of our knowledge, this is the first study exposing the potential role of structural hubs in cognitive functioning among adult glioma patients. This contribution enhances our understanding of the neurological impact of gliomas and offers insights that can be instrumental in clinical management and treatment strategies for this patient population.

In addition to examining whether a region serves as a hub, we explored the relevance of specific hubs within the network. In our study, we observed that the putamen was less frequently designated as a hub in patients compared to controls. This could mean that this hub is vulnerable to neurotoxicity. Cross-disease findings have signified this region as an important hub^77^, frequently impacted in other neurologic diseases such as multiple sclerosis^78^ and Alzheimer disease^79^. Moreover, this region has also been linked to cognitive functioning^80^. Our findings confirmed the putamen to be an important hub and showed assortativity in hubs to be positively associated with proxy IQ and attention. To investigate whether the encountered findings can be attributed to the involvement of the putamen or result from re-organization of the brain network following treatment, we performed a post-hoc subgroup analysis in patients without tumoral involvement of the putamen, showing similar findings. This implies that our results more likely represent reorganization of the brain network rather than be driven by the lesion location in the putamen. However, due to the small sample size, results must be interpreted with caution. We suggest future studies to investigate more in-depth the vulnerability of the putamen after neurotoxic treatments and its role in cognitive functioning.

The precise mechanism by which these hubs are damaged, and the respective contributions of glial tumor cells and their treatment, remain topics of ongoing debate. One﻿ plausible hypothesis regarding the specific mechanism of hub-related failure is the "hub overload and failure theory", which has garnered increasing attention in recent years. According to this theory, overloading functional hubs beyond a certain threshold, coupled with an increase in connectivity, may ultimately result in a catastrophic system collapse. Support for this hypothesis primarily stems from evidence in functional network studies conducted in the context of other diseases, i.e. in Alzheimer’s disease^75^ and schizophrenia^63^. This concept may also extend to the context of increased structural connectivity, where heightened information flow results from the rewiring of white matter tracts. Hubs that receive numerous white matter connections are densely populated with synapses, placing a substantial metabolic demand on supporting astrocytes ^81^. This heightened metabolic demand has the potential to lead to system collapse. We found higher assortativity in hubs, which was associated with cognitive functioning more than within non-hubs. The inefficient rewiring of hubs might have played a role in overloading and subsequent collapse of these hubs and their coherence, resulting in lower assortativity and potentially contributing to cognitive impairment. In﻿ the case﻿ of gliomas, it is worth noting that hubs have already been identified as preferential locations for tumor occurrence^14^. Furthermore, glioma occurrence was higher in regions with greater local and integrative connectivity (especially in high-grade tumors)^14,82,83^. This observation of hyperexcitability and hyperconnectivity of hubs further emphasizes the significance of understanding how these hubs, their connectivity, and metabolic demands may contribute to the pathophysiology of tumor growth and network damage. Another potential explanation could be the hub-specific damage resulting from radiotherapy, possibly following a dose–response relationship. The vulnerability of hubs to neurotoxic events has been previously documented^17^. Small-sample studies have previously established a link between the radiation dose and hub-specific cognitive decline, yielding compelling evidence for this hypothesis^31,35^. However, it’s important to acknowledge that the existing evidence on this topic is still limited. Additionally, the nature of the dose–response relationship, such as whether damage occurs after a certain threshold dose or increases with an increasing dose, remains to be thoroughly investigated. It would be valuable to conduct a more in-depth exploration of the vulnerability of hubs to ionizing radiation. Moreover, including a more diverse population regarding treatment and tumor location is recommended to disentangle the individual impact of each treatment component (i.e. surgery, chemotherapy, and radiotherapy). This will provide a more comprehensive understanding of the complex interplay between these factors and their effects on the brain’s network architecture in patients with gliomas.

Inconsistent with our hypotheses, betweenness centrality (in hubs) and nodal strength (in hubs and non-hubs) of the structural networks was not statistically different between groups. A general network adaptation, as suggested by changes in the abovementioned four graph measures, may indirectly have contributed to normalization of local centrality and nodal strength across the nodes of the network. Lack of these findings could also be due to an insufficient sample size to detect a potential smaller group difference. Larger sample size studies are warranted to determine whether these metrics are indeed less affected in glioma or if there may be another compensatory mechanism at play, which could explain the observed minor changes in this patient population.

### Strengths

When conducting graph theory analyses, various methodological decisions such as edge definition and weighting, normalization procedures, parcellation schemes, and (non-)hub definitions can have a significant impact on the graph measure being studied^84^. In this study, we used state of the art diffusion-weighted sequences, which allowed applying most recent processing techniques, including multi-shell multi-tissue CSD, anatomically-constrained tractography and SIFT2-reweighting. These techniques enabled us to incorporate the contribution of various cerebral tissues, to model crossing fibers and to enhance the precision of the white matter tract estimations. Furthermore, by using virtual brain grafting^50^ we prevented the potential loss of tract modellings in patients with extensive lesions, thereby minimizing the loss of valuable patient data.

Additional strengths of the study encompass a substantial sample size and comprehensive multimodal assessment within a relatively rare population. Furthermore, the inclusion of a control group matched for age, education and sex enabled meaningful comparisons between groups, namely whether alterations in structural topology were evident within the survivor group and whether these differences underpinned the disparities in cognitive performance observed between the two groups.

### Limitations and future perspectives

Some variables collected in this study exhibited non-normality, e.g. w-scores of memory due to a ceiling effect of the Hopkins Verbal Learning Test-Revised. In this context, both non-parametric tests were used, such as the Mann–Whitney U test, either log-transformation of the data was performed to be able to execute the ANOVA model.

While we identified significant associations between graph measures and cognitive outcomes, it is important to note that graph measures of local efficiency and clustering coefficient are related while global efficiency and characteristic path length are inversely related. Hence, the fact that we found group differences in all of these measures, could partly be explained by their interdependencies. Nevertheless, post-hoc correlations of respectively r = 0.563 and r = − 0.782 indicate that these measures are not perfectly interchangeable, and contain complementary information.

Moreover, although we maximally corrected for the EPI distortion induced by the glial tumor and its treatment by implementing virtual brain grafting^50^ and EPI distortion correction^57^ during the pre-processing steps, we cannot dismiss the possibility that the diffusion signal in the vicinity of the tumor may have impacted our results. This is a limitation which is inherent to the patient population.

Similarly, we cannot exclude the possible impact of the selection of the atlas on our findings. Future studies could further test the impact of atlas selection itself on damaged brain networks as investigated in this study.

Furthermore, it is worth noting that glioma patients most commonly presented with frontal lesions, which could potentially be linked to hub regions. We cannot exclude a potential bias introduced by the selection of our patient population.

Additionally, we did not analyze the associations between the nodal graph measures that did not differ between groups in both hubs and non-hubs (betweenness centrality and nodal strength) and cognitive outcomes. Therefore, we cannot make assumptions for these graph measures. For the graph measures that did differ between groups, we additionally checked the correlations between nodal measures and cognitive outcomes in each group separately. The patient group showed larger heterogeneity, most likely due to technical (VBG) and clinical variability (tumor volume/location).

Moreover, in our mixed-design ANOVA model, no clinical factors were included. In a post-hoc analysis, we reperformed the analyses while controlling for age and intracranial volume, showing robust results. No significant effect of tumor volume or hemisphere was observed in our patient cohort. However, we recognize that other factors, such as tumor location, grade, IDH1 mutation, edema, and the use of anti-epileptic drugs^30,85,86^ may influence cognitive outcomes and we therefore suggest that larger sample studies should further explore such a more comprehensive model.

## Conclusion

Glial tumor cells engage in extensive communication with each other and the surrounding brain network. Alterations in the structural brain network topology after glioma treatment become clinically meaningful once patients demonstrate a functional impact in daily life.

In this study, we identified associations between cognitive functioning and nodal graph measures, particularly within hubs. Specifically, we found that assortativity within hubs played a crucial role. Structural nodal hub-related graph metrics are thus important to consider when predicting cognitive outcomes in glioma survivors, as they may offer valuable insights for clinical management and treatment strategies in this patient group.

### Supplementary Information

Supplementary Information.

## Data Availability

The data that support the findings of this study are available from the corresponding author, upon reasonable request. The code is available on the Open Science Framework (https://osf.io/7t3xu/).
