# Supplementary material for: Brain network topology and its cognitive impact in adult glioma survivors
Source: Sci Rep. 2024 Jun 4;14:12782. doi: 10.1038/s41598-024-63716-2 (PMC11150467; doi:10.1038/s41598-024-63716-2)

Brain network topology and its cognitive impact in adult glioma survivors

Laurien De Roeck*[1,2], Jeroen Blommaert[2,3], Patrick Dupont[3,4], Stefan Sunaert[3,5], Charlotte Sleurs[2,6] and Maarten Lambrecht[1,2,3]

Author affiliations:

1 Department of Radiation-Oncology, University Hospitals Leuven, Belgium

2 Department of Oncology, KU Leuven, Belgium

3 Leuven Brain Institute, KU Leuven, Belgium

4 Department of Neurosciences, KU Leuven, Belgium

5 Department of Imaging and Pathology, KU Leuven, Belgium

6 Department of Cognitive Neuropsychology, Tilburg University, the Netherlands

Supplementary Materials

Methods: imaging processing pipeline

Software links:

- Automatic tumor segmentation:
  - Resseg: https://github.com/fepegar/resseg
  - HD-glio-auto: https://github.com/NeuroAI-HD/HD-GLIO
  - Fastsurfer: https://github.com/deep-mi/FastSurfer
- ITKsnap (manual correction lesions): http://www.itksnap.org/pmwiki/pmwiki.php
- Virtual Brain Grafting: https://github.com/KUL-Radneuron/KUL_VBG
- ANTs: https://github.com/ANTsX/ANTs
- MRTrix: https://mrtrix.readthedocs.io/en/latest/
- Brain Connectivity Toolbox: https://www.brain-connectivity-toolbox.net

Results

Mixed-design ANOVA (within-subjects)

Participant groups exhibit significant differential nodal strength across the within-subject hub nodes ($F(5.485, 537.550)=8.782$, $p<.001$, $\eta p^2 =.082$) and non-hub nodes ($F(11.667,1143.318)= 2.929$, $p=.0177$, $\eta p^2 =.029$), with higher nodal strength in hubs (mean=.359(.003), 95% CI:.355,.363) compared to non-hub regions (mean=-.396(.004), 95%CI: -.403,-.388), demonstrating significant interaction effects ($p=.020$) rather than mere average differences.
Moreover, the data yielded that participant groups exhibit significant differential betweenness centrality across the within-subject nodes, in hubs ($F(4.057,397.578)=9.391$, $p<.001$, $\eta p^2=.087$) and in non-hubs ($F(12.504,3.403)=15.794$, $p<.001$, $\eta p^2=.148$).
Across the within subject nodes, shortest path length differed significantly in hubs ($F(5.902,499.013)=7.215$, $p<.001$, $\eta p^2=.069$) but not in non-hubs ($F(13.6,1305)=1.931$, $p=.250$, $\eta p^2=.020$). Finally, both in hubs and non-hubs local efficiency, assortativity and clustering coefficient differed significantly across within-subject nodes (see Supplementary Table S4).

Supplementary Tables

Table S1 Hub regions and likelihood of being a hub across groups

Hubs	Patients (%)	Controls (%)	One-sided *p*
Left superior frontal	98	100	0.500
Right superior frontal	100	100	-
**Left putamen**	66	96	<0.001*
**Right putamen**	81	96	0.046*
Left precentral	100	100	-
Right precentral	100	100	-
Left postcentral	94	100	0.121
Right postcentral	100	94	0.121
Left thalamus	100	100	-
Right thalamus	100	100	-
Left superior parietal	78	90	0.086
Right superior parietal	80	96	0.06

*: significant value, with threshold of $p<.05$

Table S2 Overview of pure non-hubs

Pure non-hub	Patients	Controls
Left entorhinal		
fusiform		
Inferior temporal		
lingual		
medialorbitofrontal		
parahippocampal		
**paracentral**		
parsopercularis		
**parsorbitalis**		
parstriangularis		
pericalcarine		
Rostral anterior cingulate		
transverse temporal		
insula		
amygdala		
accumbens area		
Right amygdala		
accumbens area		
**entorhinal**		
fusiform		
**isthmus cingulate**		
lingual		
medialorbitofrontal		
parahippocampal		
**paracentral**		
parsorbitalis		
parstriangularis		
**pericalcarine**		
**posterior cingulate**		
Rostral anterior cingulate		
transverse temporal		
insula		

Pure non-hub (hub score of 0 in all participants of the defined group) in patients compared to controls indicated in grey. Nodes which are defined as pure non-hub in controls but not in patients are indicated in bold, while nodes which are defined as pure non-hub in patients but not in controls are indicated in italic.

Table S3 Mean and SD of graph measures over nodes and subjects per group

Nodal graph measure	Participant group	Node group	Mean	SD
**Local efficiency**	Patients	Hubs	47.11	11.70
		Non-hubs	4.72	2.17
	Controls	Hubs	36.40	6.90
		Non-hubs	3.11	0.66
**Assortativity**	Patients	Hubs	2.35	0.22
		Non-hubs	-3.11	0.48
	Controls	Hubs	3.07	0.15
		Non-hubs	-3.65	0.24
**Clustering coefficient**	Patients	Hubs	1.54	0.11
		Non-hubs	2.75	0.20
	Controls	Hubs	1.36	0.06
		Non-hubs	2.43	0.13
**Nodal strength**	Patients	Hubs	2.38	0.12
		Non-hubs	0.50	0.05
	Controls	Hubs	2.41	0.08
		Non-hubs	0.47	0.02
**Betweenness centrality**	Patients	Hubs	9.03	1.10
		Non-hubs	0.09	0.05
	Controls	Hubs	9.19	0.95
		Non-hubs	0.16	0.08
**Shortest path length**	Patients	Hubs	0.99	0.07
		Non-hubs	1.70	0.13
	Controls	Hubs	0.91	0.03
		Non-hubs	1.62	0.06

Table S4 Within-subjects ANOVA results

Nodal graph measure	*Node group*	*df*	*df (error)*	*F-value*	*p-value*[a]	*ηp²*
Local efficiency	Hubs	5.244	513.942	.519	.005	.044
	Non-hubs	13.455	1318.618	301.150	.000	.754
Assortativity	Hubs	5.338	523.093	11.744	<.001	.107
	Non-hubs	8.664	788.452	.228	001	.064
Clustering coefficient	Hubs	6.593	646.09	3.819	.007	.038
	Non-hubs	13.216	1295.18	3.182	.002	.031
Nodal strength	Hubs	5.485	537.550	8.782	<.001	.082
	Non-hubs	11.667	1143.318	2.929	.0177	.029
Betweenness centrality	Hubs	4.057	397.578	9.391	<.001	.087
	Non-hubs	12.504	3.403	15.794	<.001	.148
Shortest path length	Hubs	5.902	499.013	7.215	<.001	.069
	Non-hubs	13.6	6,1305	1.931	.250	.020

[a]*Bonferroni-corrected*

Table S5 Mixed-design ANOVA correcting for covariates ICV & age

Nodal graph measure	*Node group*	*df*	*df (error)*	*F-value*	p_{bonf}
Local efficiency	Hubs	1	98	30.948	<.001
	Non-hubs	1	98	26.688	<.001
Assortativity	Hubs	1	98	51.518	<.001
	Non-hubs	1	98	48.830	<.001
Clustering coefficient	Hubs	1	98	115.541	<.001
	Non-hubs	1	98	96.495	<.001
Nodal strength	Hubs	1	98	3.049	.084
	Non-hubs	1	98	13.315	<.001
Betweenness centrality	Hubs	1	98	.942	.334
	Non-hubs	1	98	37.215	<.001
Shortest path length	Hubs	1	98	64.997	<.001
	Non-hubs	1	98	11.220	.001

Table S6 Associations between nodal graph measures and cognitive domain scores

Nodal measure	*Node*	*Hub (H) or non-hub (NH)*	*Memory*	*Executive function*	*Attention*	*Motor functioning*	*Language*	*Proxy IQ*
Clustering coefficient	Right superior frontal	H					r=-.414 (p<.001)	
Local efficiency	Right superior frontal	H					r=.400 (p=.002)	
	Right entorhinal	NH		r=.534(p<.001)				
	Right fusiform	NH	r=.555(p<.001)					
	Right parsorbitalis	NH		r=.714(p<.001)				
	Right pericalcarine	NH			r=.445(<.001)	r=.450 (p<.001)		
	Right rostral anterior cingulate	NH					r=.675 (p<.001)	
Shortest path length	Left thalamus	H						r=-.400(p=.002)
	Right thalamus	H						r=-.405
	Right superior frontal	H						r=-.4 (p=.002)
	Right entorhinal	NH		r=-.447(p<.001)				
	Right parsorbitalis	NH		r=-.713(p<.001)				
	Right pericalcarine	NH			r=-.515 (p<0.001)			
	Right rostral anterior cingulate	NH					r=-.549(p<.001)	r=-.400(p=.001)
Assortativity	Left postcentral	H			r=.463 (p<.001)			r=.638(p<.001)
	Left precentral	H			r=.502 (p<.001)			r=.549(p<.001)
	Left superior frontal	H			r=.526(p<.001)			r=.491 (p<.001)
	Left superior parietal	H			r=.525 (p<.001)			r=.776 (p<.001)
	Left thalamus	H			r=.529(p<.001)			r=.758(p<.001)
	Left putamen	H			r=.573(p<.001)			r=.805(p<.001)
	Right thalamus	H			r=.573 (p<.001)			r=.695(p<.001)
	Right putamen	H			r=.611(p<.001)			r=.794(p<.001)
	Right postcentral	H			r=.544(p<.001)			r=.733(p<.001)
	Right precentral	H			r=.608(p<.001)			r=.588(p<.001)
	Right superior frontal	H			r=.522 (p<.001)			r=.488(p<.001)
	Right superior parietal	H			r=.488(p<.001)			r=.761(p<.001)
	Left inferior temporal	NH						r=.449(p<.001)
	Left lingual	NH				r=.505(p<.001)		
	Left rostral anterior cingulate	NH					r=.542(p<.001)	
	Left insula	NH						r=.454(p<.001)
	Left accumbens area	NH						r=-.507(p<.001)
	Right entorhinal	NH		r=.829 (p<.001)				
	Right fusiform	NH	r=.853(p<.001)					
	Right isthmus cingulate	NH					r=.425(p<.001)	
	Right pericalcarine	NH			r=.7(p<.001)	r=.42(p<.001)		
	Right posterior cingulate	NH					r=.449(p<.001)	
	Right rostral anterior cingulate	NH					r=.985(p<.001)	

Pearson correlations of nodal graph measures of clustering coefficient, local efficiency, shortest path length and assortativity with w-scores per cognitive domain. Only significant Bonferroni-corrected values are displayed

Table S7: Frequency of significant correlations of cognitive outcomes in hubs versus non-hubs.

Graph measure	Frequency of correlation within hubs (%)	Frequency of correlation within non-hubs (%)	p-value
**Clustering coefficient**	8.33	0	.24
**Local efficiency**	8.33	17	.99
**Shortest path length**	25	13.33	.143
**Assortativity**	100	36.67	<.001*

The McNemar's test was used to statistically test for group differences between hubs versus non-hub. P-values are Bonferroni corrected.* significant result.

Supplementary Materials

Post-hoc analyses: binary networks and non-normalized graph measures

We recomputed the analyses for binary graphs at different densities, but we have to keep in mind that binary networks with low density are not reproducible. Furthermore, by keeping the density the same between patients, we may also introduce spurious connections. By using weighted graphs with no or soft thresholding, we indeed also take spurious connections into account but the lower weights in that case, diminish the effect of these connections.

1. **Hub identification** (hubscore ≥2 in >80% healthy controls)

In weighted networks, we identified 12/78 nodes as hub regions. In binary networks, we identified 9-14 hubs (for densities of 10%: n=9, 20%: n=12, 30%: n=14), which largely overlap with the hubs identified in the weighted networks. In weighted networks, the postcentral gyrus was identified as a hub, but not in the binary networks, while the precuneus was identified as a hub in the binary networks but not in the weighted networks.

Table S8: percentage of healthy controls with hubscore ≥ 2

Node	Weighted networks	Binary networks for different densities				
		10%	15%	20%	25%	30%
ctx-lh-postcentral	100	4	2	16	14	6
ctx-lh-precentral	100	98	98	92	74	72
ctx-lh-precuneus	44	70	94	100	100	100
ctx-lh-superiorfrontal	100	100	100	100	100	100
ctx-lh-superiorparietal	90	66	84	88	90	92
ctx-lh-superiortemporal	38	70	84	98	98	92
Left-Thalamus	100	100	100	100	100	100
Left-Putamen	96	92	100	98	100	100
Left-Pallidum	12	10	32	52	68	80
Right-Thalamus	100	100	100	100	100	100
Right-Putamen	96	92	100	100	100	100
Right-Pallidum	8	10	20	44	68	84
ctx-rh-postcentral	94	2	2	6	10	4
ctx-rh-precentral	100	100	94	74	82	74
ctx-rh-precuneus	68	90	100	100	100	100
ctx-rh-superiorfrontal	100	100	100	100	100	100
ctx-rh-superiorparietal	96	74	98	98	100	96
ctx-rh-superiortemporal	12	64	60	70	88	92

Ctx= cortex; lh= left; rh=right

2. Whole brain graph measures (non-parametric Mann Whitney-U tests)

2.1 Weighted graph measures (no normalization)

In patients compared to HC:

- lower clustering coefficient (p= 1.2509e-14)
- higher local efficiency (p= 1.1851e-14)
- higher characteristic path length (p=0.87)
- higher global efficiency (p=0.0015)

2.2 Binary networks

In patients compared to HC:

- Higher clustering coefficient: density 5% (p = 6.03E-02), density 10% (p = 7.91E-01), density 20% (p = 6.12E-01), density 25% (p = 1.71E-01), density 30% (p = 1.17E-01), density 35% (p = 2.62E-02), density 40% (p = 5.65E-01)
- Lower clustering coefficient: density 15% (p = 2.06E-01)
- higher local efficiency: density 5% (p = 1.14E-01), density 10% (p = 7.38E-01), density 30% (p = 3.16E-01), density 35% (p = 8.30E-02)
- Lower local efficiency: density 15% (p = 2.24E-01), density 20% (p = 9.70E-01), density 25% (p = 6.08E-01), density 40% (p = 9.86E-01)
- Higher characteristic path length: density 15% (p = 7.06E-12), density 20% (p = 8.38E-12), density 25% (p = 1.36E-09), density 30% (p = 1.92E-08), density 35% (p = 3.42E-07), density 40% (p = 5.78E-06)

3. Nodal graph measures

Binary networks

Table S9: Mixed-design ANOVA between-subjects (density 10%)

Nodal graph measure	*Node group*	*df*	*df (error)*	*F-value*	p_{bonf}
Local efficiency	Hubs	1	98	2.696	.104
	Non-hubs	1	98	1.055	.307
Clustering coefficient	Hubs	1	98	1.934	.167
	Non-hubs	1	98	.246	.621
Nodal strength	Hubs	1	98	73.836	<.001*
	Non-hubs	1	98	37.142	<.001*
Betweenness centrality	Hubs	1	98	.424	.516
	Non-hubs	1	98	32.263	<.001*
Shortest path length	Hubs	1	98	81.518	<.001*
	Non-hubs	1	98	9.595	.003*

Note: assortativity not computable and reliable for these low densities

Table S10: Mixed-design ANOVA between-subjects (density 20%)

Nodal graph measure	*Node group*	*df*	*df (error)*	*F-value*	p_{bonf}
Local efficiency	Hubs	1	98	.027	.870
	Non-hubs	1	98	26.688	<.001*
Assortativity	Hubs	1	98	32.118	<.001*
	Non-hubs	1	98	.592	.444
Clustering coefficient	Hubs	1	98	1.519	.221
	Non-hubs	1	98	.041	.839
Nodal strength	Hubs	1	98	68.686	<.001*
	Non-hubs	1	98	33.444	<.001*
Betweenness centrality	Hubs	1	98	.976	.326
	Non-hubs	1	98	16.784	<.001*
Shortest path length	Hubs	1	98	67.764	<.001*
	Non-hubs	1	98	7.676	.007*

Table S11: Mixed-design ANOVA between-subjects (density 30%)

Nodal graph measure	*Node group*	*df*	*df (error)*	*F-value*	p_{bonf}
Local efficiency	Hubs	1	98	4.904	.029*
	Non-hubs	1	98	.051	.822
Assortativity	Hubs	1	98	48.206	<.001*
	Non-hubs	1	98	50.643	<.001*
Clustering coefficient	Hubs	1	98	6.138	.015*
	Non-hubs	1	98	.618	.434
Nodal strength	Hubs	1	98	93.888	<.001*
	Non-hubs	1	98	54.620	<.001*
Betweenness centrality	Hubs	1	98	18.199	<.001*
	Non-hubs	1	98	7.069	.009
Shortest path length	Hubs	1	98	79.077	<.001*
	Non-hubs	1	98	.022	.883

Table S12: Mixed-design ANOVA between-subjects (density 40%)

Nodal graph measure	*Node group*	*df*	*df (error)*	*F-value*	p_{bonf}
Local efficiency	Hubs	1	98	12.242	<.001*
	Non-hubs	1	98	.051	.822
Assortativity	Hubs	1	98	48.206	<.001*
	Non-hubs	1	98	50.643	<.001*
Clustering coefficient	Hubs	1	98	12.580	<.001*
	Non-hubs	1	98	.618	.434
Nodal strength	Hubs	1	98	76.630	<.001*
	Non-hubs	1	98	54.620	<.001*
Betweenness centrality	Hubs	1	98	40.669	<.001*
	Non-hubs	1	98	7.069	.009*
Shortest path length	Hubs	1	98	71.130	<.001*
	Non-hubs	1	98	.022	.883

In conclusion, as the densities of the binary networks increased, we observed a convergence in the results of the binary networks towards those of the weighted networks. While significant differences were predominantly identified in the weighted networks and binary networks with higher network densities (30-40%), between-group disparities in local efficiency, clustering coefficient, and path length were more frequently detected in the weighted networks. Conversely, differences in nodal strength between groups were not evident in the weighted networks, whereas they were observed in the binary networks.

4. Correlations between nodal measures and cognitive outcomes for the binary networks (density of 40%)

Table S13: Correlations between graph measures calculated baed on binary network with density of 40% and cognitive outcomes per domain

Nodal measure	*Node*	*Hub*	*Memory*	*Executive function*	*Attention*	*Motor function*	*Language*	*Proxy IQ*
Clustering coefficient	Right superior frontal	H					r=-.400 (p<.001)	
Local efficiency	Right superior frontal	H					r=.400 (p<.001)	
	Right rostral anterior cingulate	NH					r=.320 (p=.003)	
Shortest path length	Left thalamus	H						r=-.295(p=.004)
	Right thalamus	H						r=-.234 (p=.024)
	Right superior frontal	H						r=-.254(p=.032)
	Right entorhinal	NH		r=-.400(p<.001)				
	Right parsorbitalis	NH		r=-.671(p<.001)				
	Right parstriangularis	NH						r=-.380 (p<.001)
	Right rostral anterior cingulate	NH					r=-.458(p<.001)	r=-.390(p<.001)
Assortativity	Left precentral	H			r=.234 (p=.024)			r=.340(p=.001)
	Left superior frontal	H			r=.230(p=.030)			r=.360 (p=.001)
	Left thalamus	H						r=.460(p<.001)
	Left putamen	H						r=.400(p<.001)
	Right thalamus	H						r=.695(p<.001)
	Right putamen	H						r=.794(p<.001)
	Right precentral	H						r=.421(p<.001)
	Right superior frontal	H						r=.401(p<.001)
	Left inferior temporal	NH						r=.290(p=.033

Left lingual	NH				r=.320p=.007)		
Left rostral anterior cingulate	NH					r=.300(p=.005)	
Left insula	NH						r=.400(p<.001)
Right isthmus cingulate	NH					r=.232(p=.049)	
Right pericalcarine	NH			r=.335(p=.014)			
Right posterior cingulate	NH					r=.321(p=.004)	
Right rostral anterior cingulate	NH					r=.512(p<.001)	

These correlations, although slightly diminished, remain robust. Regarding local efficiency, only one significant correlation persists within the non-hubs, while the correlations within the hubs remain unchanged. Similarly, for shortest path length, the correlations remain consistent within the hubs, but there is a variation within the non-hubs (e.g., the right pars triangularis exhibits a significant association instead of the right pericalcarine). In terms of assortativity, most correlations with IQ persist, although the associations with attention are less distinct in the binary networks. Additionally, fewer significant associations were observed between assortativity of hub nodes and cognitive outcomes (8 nodes instead of 11).

Supplementary Figures

Figure S1: Correlation matrices of cognitive outcomes (all subjects)

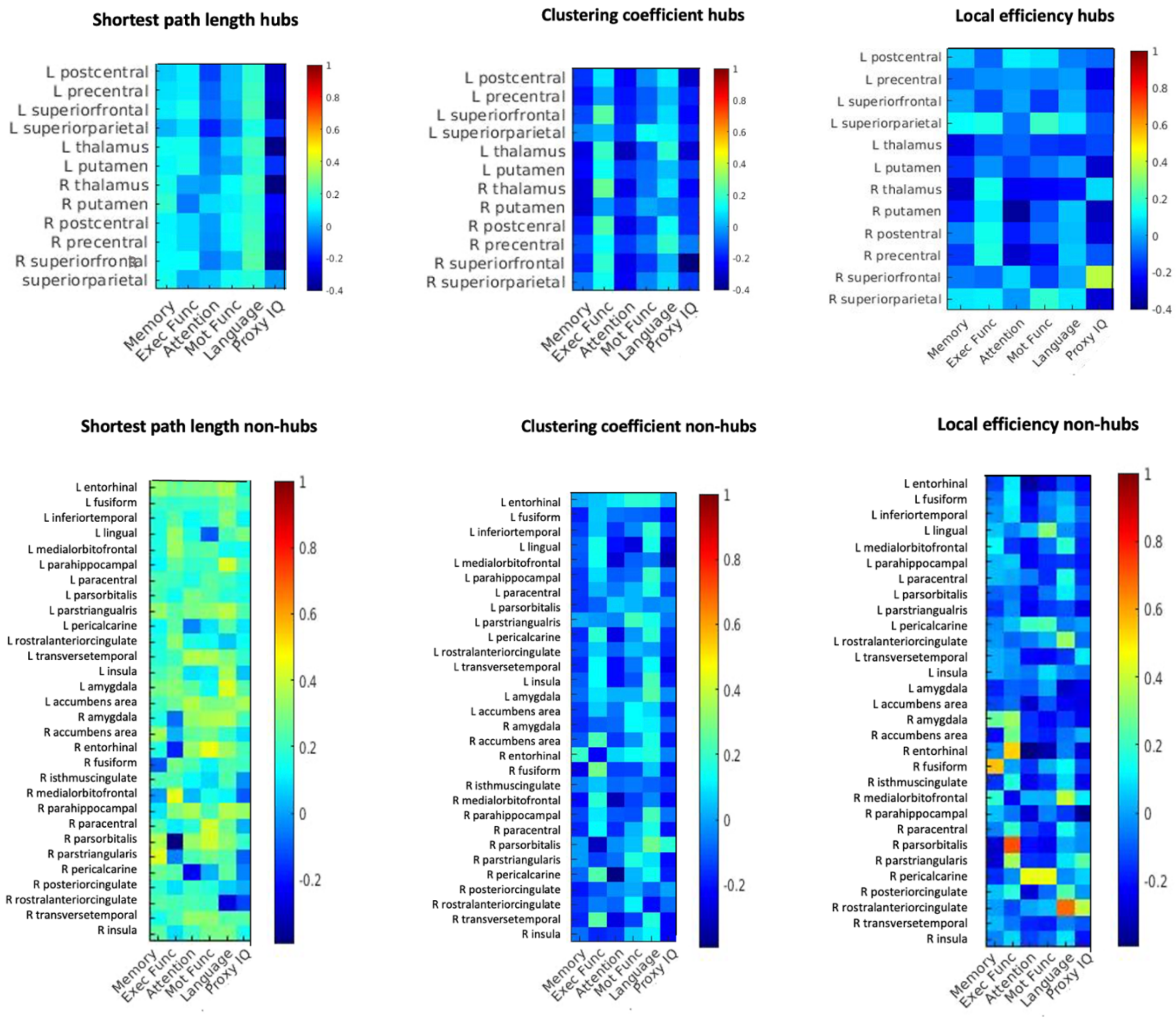

Correlation matrices of cognitive outcomes (n=6) per node in hubs and non-hubs for nodal measures of shortest path length, clustering coefficient & local efficiency

Figure S2: Correlation matrices of cognitive outcomes per group in hubs (patients and controls)

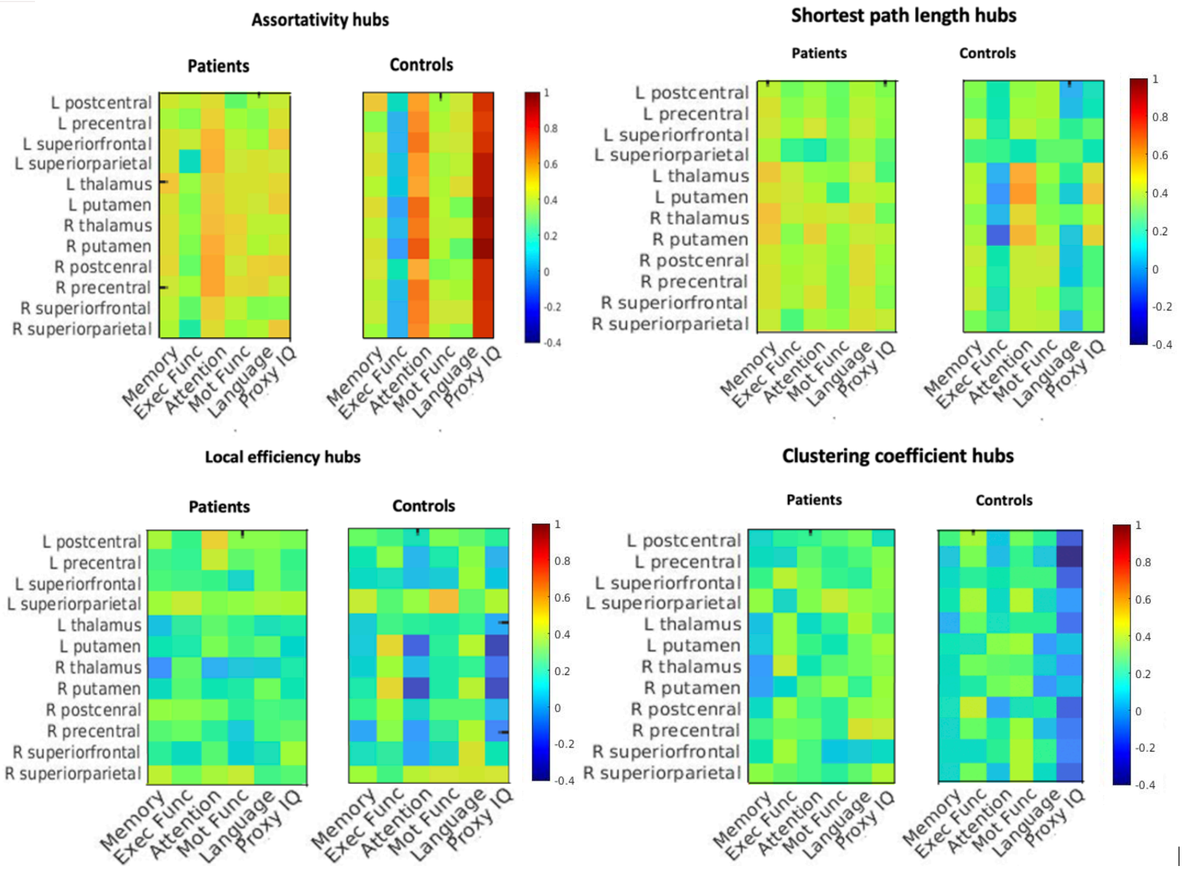

Figure S3: Correlation matrices of cognitive outcomes per group in non-hubs (patients and controls)

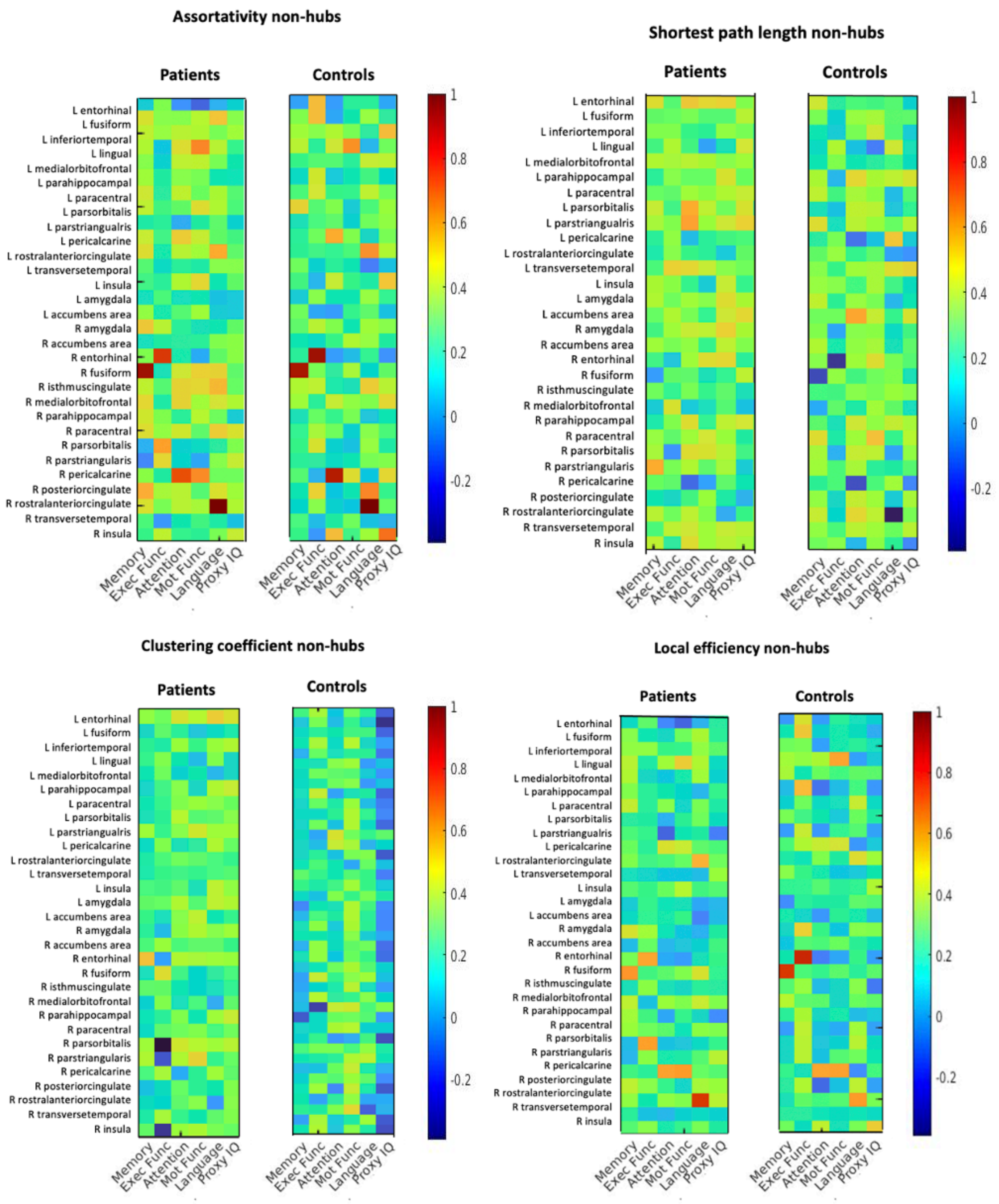

Supplement: Supplementary file 1 — Supplementary Information. [file 41598_2024_63716_MOESM1_ESM.pdf]
